# Design of CIAO, a research program to support the development of an integrated approach to prevent overweight and obesity in the Netherlands

**DOI:** 10.1186/2052-9538-1-5

**Published:** 2014-02-19

**Authors:** Marije TM van Koperen, Rianne MJJ van der Kleij, Carry CM Renders, Matty MR Crone, Anna-Marie AM Hendriks, Maria M Jansen, Vivian VM van de Gaar, Hein JH Raat, Emilie ELM Ruiter, Gerard GRM Molleman, Jantine AJ Schuit, Jacob JC Seidell

**Affiliations:** Faculty of Earth and Life Sciences, Institute of Health Sciences, VU University Amsterdam, De Boelelaan 1085, 1081 HV Amsterdam, The Netherlands; Department of Public Health & Primary Care, Leiden University Medical Centre, PO Box 9600, 2300 RC Leiden, The Netherlands; Health Sciences, University Maastricht, PO Box 616, 6200 MD Maastricht, The Netherlands; Caphri research School for Public Health and Primary care, PO Box 616, 6200 MD Maastricht, The Netherlands; Public Health, Erasmus University Medical Center, PO Box 2040, 3000 CA Rotterdam, The Netherlands; Department of Primary and Community Care, Academic Collaborative Center AMPHI Integrated Health Policy, Radboud University Medical Centre, PO Box 9101, 6500 HB Nijmegen, The Netherlands; National Institute for Public Health and the Environment (RIVM), PO Box 1 3720, BA Bilthoven, The Netherlands; Erasmus University Medical Center, child and adolescent public health, PO Box 2040, 3000 CA Rotterdam, The Netherlands

**Keywords:** Integrated approach, Overweight, Community, Political support, Social marketing, Parenting, Implementation, Evaluation framework

## Abstract

**Background:**

The aim of this paper is to describe the research aims, concepts and methods of the research Consortium Integrated Approach of Overweight (CIAO). CIAO is a concerted action of five Academic Collaborative Centres, local collaborations between academic institutions, regional public health services, local authorities and other relevant sectors in the Netherlands. Prior research revealed lacunas in knowledge of and skills related to five elements of the integrated approach of overweight prevention in children (based upon the French EPODE approach), namely political support, parental education, implementation, social marketing and evaluation. CIAO aims to gain theoretical and practical insight of these elements through five sub-studies and to develop, based on these data, a framework for monitoring and evaluation.

**Methods/Design:**

For this research program, mixed methods are used in all the five sub-studies. First, problem specification through literature research and consultation of stakeholders, experts, health promotion specialists, parents and policy makers will be carried out. Based on this information, models, theoretical frameworks and practical instruments will be developed, tested and evaluated in the communities that implement the integrated approach to prevent overweight in children. Knowledge obtained from these studies and insights from experts and stakeholders will be combined to create an evaluation framework to evaluate the integrated approach at central, local and individual levels that will be applicable to daily practice.

**Discussion:**

This innovative research program stimulates sub-studies to collaborate with local stakeholders and to share and integrate their knowledge, methodology and results. Therefore, the output of this program (both knowledge and practical tools) will be matched and form building blocks of a blueprint for a local evidence- and practice-based integrated approach towards prevention of overweight in children. The output will then support various communities to further optimize the implementation and subsequently the effects of this approach.

## Background

Childhood overweight (and obesity) is one of the most serious public health challenges of the twenty-first century in the world [1]. In the Netherlands, the number of overweight children increased sharply in the last decade. In 2010, more than 14% of Dutch children aged between 2 and 21 were overweight, of which almost 2% were obese [2]. To stabilize or decrease the current prevalence of overweight, it is widely accepted that interventions should be comprehensive, targeted at multiple levels, address the drivers of overweight and should be directed at children and their environment [3–10]. In this paper we will refer to such comprehensive programs as the 'integrated approach’.

The prevalence rates and the severity of overweight, especially regarding complications associated with obesity, put it high on the political and public health agenda of policy makers and funding agencies in the Netherlands. They are becoming increasingly aware that an integrated approach might be the only sustainable solution to this so-called wicked problem of overweight. A wicked problem is defined as a complex problem that prevails in society, with multiple interwoven determinants and for which evidence for the effectiveness of potential solutions is often lacking [11]. Driven by the urgency of tackling this extensive and serious public health problem and the growing awareness that the integrated approach might be the only sustainable solution, multiple Dutch municipalities have initiated integrated approaches on overweight and obesity prevention in the last decade [12, 13]. Additionally, in 2009, the Dutch Ministry of Health recommended an integrated approach based upon the French EPODE program as a possible solution to tackle overweight in The Netherlands [13].

EPODE (or Together Let’s Prevent Childhood Obesity) is a French community-wide comprehensive intervention program. It aims to prevent overweight and obesity in children aged 0–12 years and their families through a multi-activity, multi-setting and multi-stakeholder approach [14, 15]. The program is coordinated at a central level. The focus is on promoting healthy behaviors regarding the importance of healthy eating and regular physical activity [14–16]. At the community-level, a project manager is nominated by local authorities. This project manager is not only trained by EPODE, but is also provided with tools and instruments that facilitate local implementation [14]. EPODE identified four critical components in its integrated approach: political commitment, public and private partnerships, social marketing and evaluation [14, 15].

**Figure 1:**
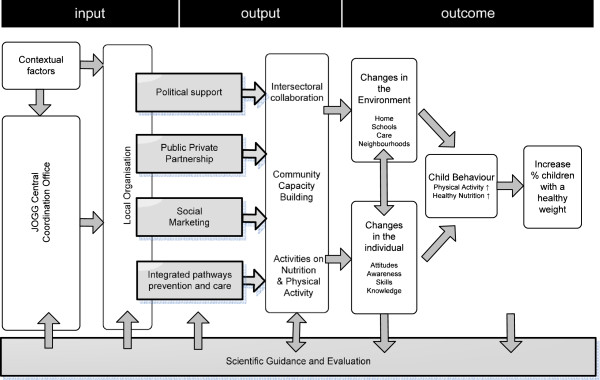
**JOGG model.** This logic model has been based upon the EPODE logic model [15]. It has been developed in the beginning of sub-study 5 by MvK, the JOGG Central Coordination Office and the first six JOGG communities. A clear difference between the two models is the starting point of the four critical components. For the Dutch situation, the developers agreed to move the critical components more to the right of the model, to the local organisation, since development and implementation of these components is mainly at local level. Also a fifth pillar was added to the JOGG model: integrated pathways prevention and care. The JOGG model is being used as a model of reference for implementation and evaluation of the local JOGG approach by JOGG Central Coordination Office and the JOGG communities. Moreover, CIAO uses this model to design and frame its research. The JOGG model has not been published previously.

It is expected that the number of municipalities in the Netherlands that implement an integrated approach will further increase in the coming years since the Minister of Health actively supported the integrated approach by the establishment of the Dutch JOGG central coordination team in 2010. JOGG stands for Youth on Healthy Weight and is a centrally coordinated and locally implemented integrated approach based on the EPODE approach. In fact, the Dutch government has set the target of the number of cities joining the JOGG programs at 75. In addition to the four critical components of EPODE, JOGG formulated a fifth component: the integrated pathways between prevention and care. The five critical components of the JOGG program are part of a logic model which is shown in Figure 1 (in grey).

To optimize the implementation of JOGG, and subsequently its effectiveness, innovative research is needed. Moreover, local health promotion specialists have indicated that they are in need of tools and guidelines to support the implementation and evaluation of this integrated approach targeting overweight and obesity [17]. However, the immediate demand for action by funders and policymakers leaves no time for thorough development of the integrated approach, such as theoretical development, qualitative testing, modelling, feasibility testing etc. Researchers have to adjust their traditional research methods to deliver knowledge and guidelines following the continuous evolution of policy and practice. Action research is specifically recommended to study such programs because it validates the dynamic processes through feedback in order to adjust the approach [18–20]. The two main functions of action research are action and evaluation. The action function is supposed to support action and to stimulate the progress of the intervention. It is assumed that this immediate feedback helps practitioners to decide how to continue, thus literally stimulating and guiding action [21]. The evaluation function seeks to monitor and ascertain processes and outcomes of interventions or actions. Such an evaluation serves to legitimize a program and increase its accountability.

### Consortium integrated approach of overweight

After the Dutch Ministry of Health had mentioned the integrated approach as a possible solution to tackle overweight [13], the research consortium CIAO was established in January 2010. This consortium consists of five Academic Collaborative Centres (ACCs) and aims to gain insight and knowledge in key-elements of the integrated approach towards overweight and obesity prevention.

An ACC is a local collaboration between 3 academic institutions, regional public health services, local authorities and other relevant sectors. Each involved ACC aims to promote knowledge exchange between municipalities, regional Public Health Services, academic public health departments and other local stakeholders on specific public health issues [22, 23]. This knowledge exchange within an ACC stimulates the translation of scientific knowledge into practical products, services and facilities [22, 24]. Moreover, it offers a unique opportunity to share processes and methodology for an effective and sustainable prevention strategy for overweight at the local level. Through collaboration, researchers can gather complementary evidence that may elucidate the picture as a whole rather than as separate and independent parts. Also the diversity of scientific, tactical and practical knowledge and skills within the ACCs can lead to cross-fertilization and new insights within CIAO. Each of the five ACCs involved in CIAO prioritize the prevention of overweight and obesity.

CIAO started with an inventory study of (inter)national interventions proven effective or promising directed at the primary prevention of overweight and obesity in children and adults and their conditions for successful implementation. Literature studies, surveys and workshops were conducted with health promotion professionals and parents in addition to interviews with experts [25]. Also more than 30 interviews were held with health promotion professionals, policymakers and researchers involved in the five ACCs. The EPODE logic model [15] was used as a framework to guide data-analysis. It appeared that many of the theoretically essential and critical elements of the EPODE approach need further definitions and operationalization [25]. For instance, 'intersectoral policy and political commitment’, 'social marketing’ and 'evaluation’ need to be further developed. Additionally, it became clear that currently a lot of potentially effective interventions have been developed to stimulate healthy dietary and physical activity behaviour in families, schools or neighbourhoods, however only a few are implemented in an appropriate and sustainable way. Furthermore, many interventions are applied in a very fragmented way. To reach an effective integrated approach, it is important to work towards more cohesion and intersectoral collaboration. It also became clear that it is necessary to further develop the role of parents in regards to their parenting skills and pedagogical knowledge within different sectors of the integrated approach. This development is especially important within the integrated approach because the participation of parents plays a central role in most interventions especially if young children are involved [26]. Finally, many sectors have indicated a need for a comprehensive evaluation framework that can be used to evaluate and monitor the processes and outcomes of the integrated approach [25].

Based on the inventory study, CIAO will continue to further develop a blueprint for a national framework of evidence-and practice-based integrated approach towards local prevention of overweight and obesity. The research program will consist of five sub-studies, conducted by five research teams integrated in the ACCs, which together will constitute the building blocks of such a blueprint. According to both JOGG and EPODE, political commitment is a critical component and is identified by CIAO as a key-element for a successful implementation of the integrated approach. Since determinants of overweight cannot only be found in the domain of public health, but also in other domains such as safety, spatial planning, economics that may influence the physical and/or social environment (more upstream determinants) [27], involvement of these responsible local government sectors is integral in changing these determinants [28]. In short, both political commitment and intersectoral collaboration between health and non-health domains are important for the success of an integrated approach [29, 30]. However, it is still not clear how this can be positively influenced [25, 29]. Therefore, the first main research question for CIAO is: *How can intersectoral collaboration between policy sectors within municipalities result in integrated policies with an effective, easy-to-implement, well-described plan of action?*

The reduction of inequalities in health is an important target in public health policies of WHO Europe and the EU. Overweight and obesity are positively correlated to low-income and low education populations, leading to a high prevalence of overweight and obesity in disadvantaged neighbourhoods [31–33]. The reach of interventions in these neighbourhoods is, however, often rather limited. To adapt or develop interventions that connect with the needs, wishes and perceptions of the population in these areas, JOGG should stimulate the use of social marketing strategies. However, the CIAO inventory study revealed that in the Netherlands, social marketing is a relatively new health promotion concept and needs further explication to fully understand the working mechanisms in order to stimulate local use and evaluation [25]. Additionally, parental skills and knowledge are key determinants of children’s behaviour. To change prevalence rates of overweight and obesity in children by improving energy-balance related behaviours, parental support is crucial [34–36]. Existing interventions in the Netherlands focus mainly on behaviour change in children and lack sufficient attention to parental support [25, 26]. This has led to the second main research question of CIAO: *How can current interventions and integrated policies be reinforced by using up-to-date parenting support, and by adaptations increasing the reach in disadvantaged neighbourhoods using social marketing strategies, resulting in effective, easy-to-implement preventive interventions?*

Moreover, it is important to gain insight into factors that influence the implementation processes of the integrated approach and interventions, especially in disadvantaged neighborhoods and into strategies to further optimize the use of these factors. Therefore, a thorough monitoring and evaluation of the implementation process is necessary, and process and effect indicators should be routinely measured. For this purpose, it is important that consensus is reached with respect to the indicators that are used to measure the progress and outcome of the integrated approach. The third study question for CIAO to answer is: *How can integrated policies be implemented in disadvantaged neighbourhoods, and how can process and effect indicators be routinely measured and applied in the development and implementation of effective local integrated policies promoting healthy weight in youth?*

In order to address these questions, CIAO has designed five sub-studies directed at the prevention of overweight and obesity in children:

1. Guiding and monitoring the process of political commitment for intersectoral collaboration leading to integrated policy,
2. Influencing reach and effect of community interventions by guiding and monitoring social marketing strategies,
3. Strengthening parenting styles and practices in existing interventions,
4. Guiding the intended adoption and implementation processes in an integrated approach,
5. Developing a theory and practice based evaluation framework.

It is essential that in each of the sub-studies several of the participating ACCs collaborate so that the consortium can optimally benefit from the vast experience and expertise available in these centres. Research will be carried out to improve the program design and implementation of JOGG as it is rolled out.

## Methods/Design

**Figure 2:**
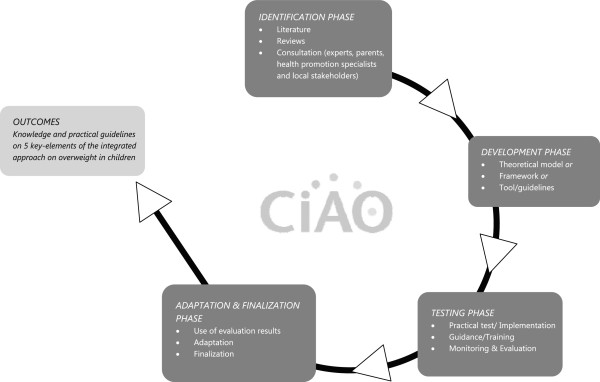
**Research outline of CIAO.** This figure provides an overview of the four research stages of all the five sub-studies of CIAO.

All five sub-studies will follow the same research cycle as shown by Figure 2. They will start with an identification phase in which the research question will be specified. In this phase, interviews will be held with experts, parents, health promotion specialists and local stakeholders, and literature search and reviews will be conducted. In the development phase, a framework, theoretical model, tool, or guidelines will be constructed based upon results from the identification phase. In the testing phase, the developed materials will be tested in practice and will be evaluated. Both quantitative and qualitative research methods will be used in this evaluation. In the adaptation and finalization phase, evaluation results from the test phase will be used to adapt and optimize the developed materials. Finally, the developed materials and gained knowledge will be the building blocks for a blueprint for a national framework of evidence-based and practice-based integrated approach towards local prevention of obesity.

The results of the five sub-studies will inform a well-rounded answer to the three main research questions. Research methodology, data-collection, data-analyses and outcomes will be matched and coordinated. To increase understanding and readability, the various sub-studies will be presented here separately (for a concise overview of the sub-studies, see Table 1).

**Table 1:** **A concise overview of the 5 sub-studies of CIAO**

Substudy nr.	Theme	ACCs	Research phases			
			Identification phase	Development phase	Testing phase	Adaptation & finalization phase
**1**	Political-administrative support	Limburg	- Literature review on operational criteria of integrated public health policies	- Develop a conceptual framework which describes the process of developing integrated public health policies.	- Apply developed conceptual framework	- Focus groups with actors at strategic and tactical levels within Dutch local governments to find solutions for previously identified barriers
			- Interviews with local governmental officials and key-informants, and theoretical reflections to gain insight in the process of developing integrated public health policies		- Interviews with local governmental officials to gain insight in hampering or facilitating factors for intersectoral collaboration	- Refinement of the developed conceptual framework based on the outcomes of the previous studies
			- Interviews with key-informants within the policy process to explore existing 'interventions for the development of integrated public health policies’		- Comparison of cases to gain insight in the effects of implementation style on interventions aimed at local governmental officials	- Refinement of the developed conceptual framework based on the outcomes of the previous studies
					Extra:	- Developing a program or policy resource that might be able to stimulate or facilitate the development of integrated public health policies
					- Test and evaluate the developed framework in Australia (NSW) - data-collection through interviews with General Managers, Directors of Community Services, Health officials and Environment and Recreation officials, and a document analysis.	
**2**	Social marketing	- CEPHIR/Eras mus/ GGD Rotterdam	- Benchmarks	- Monitoring case-studies	- Evaluation of case-studies using developed monitoring format	- Adapting monitoring tool for Dutch setting
			- Analyses of Determinants of healthy weight among children	- Develop practical monitoring tool		- Overview of determinants of applying social marketing
			- Selecting case-studies (interventions to promote healthy weight in childhood based on Social Marketing approach)			
**3**	Implementation	Noordelijk Zuid-Holland	- Systematic Review of design and quality of implementation research regarding complex integrated programs targeting overweight	- Construction of process evaluation plan & several instruments to evaluate the innovation process	- Longitudinal case-studies (5x): Interviews, questionnaires, focus groups, observational research document analyses & semi-action research social network analysis	- An overview of the level and determinants of the innovation process of the integrated approach
			- Consultation with experts and local project managers		- Parents versus teachers, the relation between task-orientation and implementation	- Guidelines/ indicators for the innovation process
						- If needed, adaptation of framework Fleuren et al [37] (for innovation process of the integrated approach)
**4**	Strengthening parenting styles and practices in existing interventions	AMPHI Nijmegen	- Analyses existing data: attitudes professionals and parents on overweight	- Development of a web-based parenting intervention (with the aim of strengthening existing overweight preventing interventions in children)	- Testing the effectiveness of this web-based intervention in a two-armed cluster randomized controlled trial	- Web-based parenting intervention to prevent overweight in children
			- Literature search of the role of parenting in interventions to prevent overweight in children	- Development of an 'local pedagogical message regarding overweight and obesity’ applicable by all local professionals working with children and their parents	- Focus groups with parents to improve the textual content of the ' local pedagogical message’	- local pedagogical message
			- Analyses of existing interventions for children and attached parental interventions		- Effect and process evaluation of implementation	
			- Focus groups to gain more insight and get specific examples of difficult daily life situations in which mothers experience problems in promoting healthy eating and physical activity among their children			
**5**	Monitoring & Evaluation	VU University Amsterdam/Windesheim University Zwolle	- Description of EPODE logic model	- Development of evaluation handbook (1.0) and evaluation tools	- Use of Evaluation Handbook (1.0) by JOGG municipalities	- Adaptation and Finalization of: evaluation handbook (2.0), evaluation planning matrix, evaluation training
			- Evaluation literature	- Development of evaluation training	- Focus groups on evaluation handbook and evaluation training	
			- Interviews with Dutch experts	- JOGG program goals and objectives	- Feedback on evaluation tools and evaluation planning matrix	
			- Comprehensive analyses of evaluation frameworks	- Development evaluation planning matrix	- Data-collection from all CIAO sub-studies inserted in evaluation framework	

### Sub-study 1: Political-administrative support

The aim of this study is to understand the process of intersectoral collaboration leading to an integrated public health policy to prevent childhood overweight and obesity. A multiple-case study design will be used, and a qualitative research approach will be adopted. In this research interviews, online questionnaires and an analysis of policy documents will be used to collect data among several local governmental organizations (i.e., our cases).

*In the identification phase,* operational criteria of integrated public health policies will be developed by using a literature review and the Behaviour Change Wheel [38] as a theoretical framework. This is required in order to analyze the policy content in the upcoming studies. Furthermore, a conceptual framework, which describes the process of developing integrated public health policies, will be developed by using interviews and theoretical reflections. Subsequently, interventions for the development of integrated public health policies will be explored by interviewing local governmental officials and key-informants within the policy making process.

Interview data will be collected in two small-sized Dutch local governments *in the development phase* to obtain insight into the factors that were hampering or facilitating intersectoral collaboration. By comparing these cases, insight into the effects of implementation style on interventions aimed at local governmental officials will be derived.

*In the testing phase,* the conceptual framework will be applied in two relatively large Dutch local governments. The aim is to explore to what extent this conceptual framework might be able to illuminate the process of developing integrated public health policies. Additionally, the definition of integrated public health policies will be used to determine if the policy content of these local governments can be considered 'integrated.’ After that, the conceptual framework will be used to evaluate the effect of a resource that was developed in New South Wales, Australia to assist local governments in developing a specific type of integrated public health policy, i.e., an active living policy. Interviews with general managers, directors of community services, health officials and environment and recreation officials and a document analysis of policies developed in the included municipalities will be used to collect data about the policy process and policy content.

*In the adaptation and finalization phase,* focus groups will be held with actors at the strategic and tactical levels within the three Dutch local governments. The focus will primarily be finding solutions for identified barriers in our previous studies.

### Sub-study 2: social marketing

Research has shown that community involvement may contribute to improved outcome effects as well as more sustainable programs with better reach and impact [16, 39–41]. It is argued that to address childhood overweight and obesity, multiple settings need to be targeted (i.e. individual, family, school and community) [42–47]. In their brief overview of community interventions and their application to the obesity epidemic, Economos and Irish-Hauser [48] conclude that “involving the community in any of the initiatives helps researchers to pinpoint the specific needs of the community, as well as to identify assets and untapped resources and solutions”. This is exactly the idea behind the use of social marketing within health promotion. This study will focus on monitoring and evaluating social marketing techniques applied in programs to promote healthy weight in childhood and to develop a monitoring tool to improve the outcomes of (parts of) programs developed with social marketing. According to French et al., social marketing strategies aim to achieve voluntary behavior change by taking the needs and wishes of the target audience as the starting point and from there, trying to understand how to best promote the desired behavior using an integrated, tailored approach [49]. Social marketing strategies aim to incorporate the community and act on an ecological level [50–53], which has led to successful examples worldwide of programs promoting healthy lifestyles among children and their families [54–58].

*In the identification phase,* public health programs aiming to prevent overweight among children in which social marketing is applied to enhance the outcomes will be explored.

*In the development phase,* a practical tool or format for monitoring social marketing will be designed This format will be based on theory, e.g. the social marketing benchmark criteria as defined by French [49] and practice, collected data from several case-studies.

*In the testing phase,* the developed monitoring tools will be tested while evaluating the application of social marketing in the case-studies using quantitative and qualitative methods.

*In the adaptation and finalization phase,* the lessons learned and insights gained from the testing phase will be used to make the practical format adaptable for practice, possibly for nationwide implementation in the Netherlands.

### Sub-study 3: strengthening parenting styles and practices in existing interventions

Following the outcomes of the inventory study of CIAO this sub-study aims to strengthen parenting styles and practices in existing interventions to prevent overweight in children. Therefore, a web-based parenting intervention, an E-learning module will be developed and tested for effectiveness. This E-learning could be added to existing interventions to prevent overweight in children and as such be an integral part of the intervention. Furthermore, a pedagogical message for parents will be developed, which can support them in preventing overweight of their child. All local professionals working with children could use this message.

*In the identification phase,* a literature search will be carried out regarding the role of parenting in the prevention of overweight in children and the involvement of parents in existing interventions. Furthermore, data from the Youth Health Care on the attitudes of professionals and parents regarding overweight in children will be analyzed. Subsequently, existing data from a large survey of parents (n=7000) on their perception of overweight and the rules parents set at home regarding healthy eating and physical (in)activity will be analyzed (data from a periodical Youth Health Care monitor). More insight into and specific examples of difficult daily life situations in which mothers experience problems in promoting healthy eating and physical activity with their child will be compiled by using focus groups comprised of mothers of different ethnic and socio-economic backgrounds. The Medical Review Ethics Committee Region Arnhem-Nijmegen approved this focus group study, reference number 2012145. This study was not liable for WMO. Written informed consent for participation in the study was obtained from participants.

*In the development phase,* the outcomes of the identification phase will be used for the development of a Dutch web-based parenting intervention. Thereafter, a Delphi method will be conducted in which we use the knowledge of different experts, researchers and professionals working with children for the development of a pedagogical message regarding overweight and obesity.

*In the testing phase,* the effectiveness of the web-based parenting intervention will be investigated in a two-armed cluster randomized controlled trial. This trial is in compliance with the Helsinki Declaration. The Medical Review Ethics Committee Region Arnhem-Nijmegen approved the study protocol, reference number 2012495, NL4280309112. This study was not liable for WMO and registered at the Dutch Trial Register NTR3938. A passive informed consent procedure will be followed in which parents (and their children) can refuse study participation. Thereafter, the usability of the textual content of the pedagogical message will be evaluated by means of focus groups with different ethnic and socio-economic backgrounds.

*In the adaptation and finalization phase,* the web-based parenting intervention and the pedagogical message will be adapted and optimized according to the findings of the testing phase, and the final versions will be disseminated.

### Sub-study 4: implementation of the integrated approach

When preventive programs are being implemented the iterative and dynamic interactions that occur often diverges from the process as originally planned. The integrated approach faces even more implementation challenges as it is based on a convoluted program plan and addresses multiple settings and involves many sectors. During the inventory study, it was concluded that there is limited knowledge of (determinants of) the implementation of the integrated approach. This lack of knowledge makes it difficult to formulate sound implementation strategies and value reported effects of the approach. Therefore, this sub-study will explore the implementation of the integrated approach and its determinants at the community level.

*In the identification phase,* experts and local project managers of several municipalities initiating the integrated approach will be consulted to identify local implementation plans and strategies to formulate a status quo. Furthermore, a systematic review will be conducted to elucidate what is already known about the implementation of the integrated approach and what instruments and outcome measures have been used to evaluate the implementation process of this approach.

*In the development phase,* a process evaluation plan will be constructed and several instruments to evaluate the implementation process of the approach will be created or adjusted. This will be guided by the information obtained during the identification phase and by the framework for determinants of innovations as formulated by Fleuren et al. [37]. The process evaluation plan will contain mixed-methods for studying the innovation process (i.e. interviews with intermediaries, observations of activities, document analysis, questionnaires, focus groups, network analysis).

*In the testing phase,* five municipalities in which a longitudinal study will be performed on the implementation process of the integrated approach will be selected. The methods for the process evaluation will be adjusted iteratively when indicated by data-collection and data-analyses.

*In the adaptation and finalization phase,* the results from the longitudinal study will be combined and compared to create an overview of the level and determinants of the implementation of the integrated approach. Interpretation of data will be based on a framework analysis of qualitative data via Atlas Ti, Qualitative Comparative Analysis, a Social Network Analysis and statistical analysis of quantitative data. Moreover, results of different analyses will be compared to triangulate our data. The process of analysis will lead to a guideline for evaluating the innovation process. Additionally, it will provide implementation indicators that could aid municipalities in formulating implementation strategies for the integrated approach. If needed, the framework of Fleuren et al. [37] will be adjusted to reflect the implementation of the integrated approach.

### Sub-study 5: scientific guidance and evaluation

This study aims to construct an evaluation framework for the integrated community approach of overweight and obesity in children in order to stimulate evaluation of JOGG. This evaluation framework will consist of an evaluation handbook set as an action plan in the planning and implementation of evaluation, supporting health promotion specialists to overcome evaluation barriers and in the meantime, build evaluation capacity. The evaluation framework will also consist of an evaluation planning matrix in which practice and evidence based knowledge from the all CIAO sub-studies will be combined. To increase understanding and readability, the methodology for the evaluation handbook will be presented first (A), followed by the description of the evaluation planning matrix (B).

#### (A) Evaluation Handbook study

*In the identification phase,* a literature study and interviews with experts, health promotion specialists and JOGG-program managers will be conducted to determine barriers in program-evaluation. Subsequently, a comprehensive search in electronic databases to identify a suitable evaluation action plan or handbook will be conducted.

*In the development phase*, the identified evaluation handbook will be translated into Dutch. Practice based examples from JOGG communities will be added to this evaluation handbook (version 1.0). Supportive educational training will be developed following the outline of the evaluation handbook. Training will follow essential aspects of the Social Cognitive Theory: modelling, practice, feedback and coaching [59].

*In the testing phase,* the evaluation handbook will be delivered to JOGG program managers to support evaluation of the local JOGG program. Educational training will be provided to the program managers and involved epidemiologists. Both the training and the evaluation handbook will be evaluated through four focus groups consisting of JOGG program managers and designated researchers and experts in community-wide intervention approaches and evaluation from research institutes and semi-governmental National Health Promoting Institutes.

Following the outcomes of the focus groups *in the adaptation and finalization phase* the handbook will be adapted and finalized.

#### (B) Evaluation planning matrix

An evaluation planning matrix is a tool that describes the evaluation questions, the indicators, data-collection instruments and time-line, data-analyses and dissemination per the main goal.

*In the identification phase,* the JOGG model will be determined. Subsequently, main goals and objectives of the JOGG-approach will be discussed and determined with the JOGG-board, JOGG central coordination office, six JOGG pilot municipalities and executive researchers of CIAO sub-studies 1,2,3 and 4. Evaluation questions, indicators and data-collection instruments will be delivered by the CIAO sub-studies 1,2,3 and 4.

In collaboration with the other CIAO researchers, *in the development phase,* these elements will be placed in an evaluation planning matrix.

*In the testing phase* the evaluation planning matrix will be submitted to experts, program managers and the JOGG central coordination office and evaluated on use, usefulness, and feasibility.

*In the adaptation and finalization phase,* the evaluation planning matrix will be adapted in accordance with results from the expert meetings and focus groups and disseminated to the JOGG central coordination office.

Both the evaluation handbook and the evaluation planning matrix will be combined in the evaluation framework for the integrated approach on overweight in children. Expert meetings will be held to create consensus and support for the evaluation framework.

## Discussion

It is generally accepted that to combat overweight and obesity, an integrated community-wide approach is needed. An inventory study showed that some elements of the integrated approach could be more important than others and that in the Netherlands these elements need further definition and operationalization [25]. The concerted research consortium CIAO is expected to contribute significantly to the understanding of these key-elements. This comprehensive study is in line with a recommendation from a recent review study to identify trends and gaps in the field of childhood obesity research done, namely the need for 'more solution-oriented research that combines individual, environmental, and policy strategies to address the problem comprehensively’ [60]. Collaborating in a research consortium in which researchers gather complementary evidence provides evidence that supports the 'whole’ picture rather than parts of it. Also, the diversity of knowledge and skills of the executive researchers and their supervisors working in the ACCs can lead to cross-fertilization that can lead to new insights. In CIAO, this will be stimulated through regular quarterly meetings attended by the executive researchers, their supervisors, the steering committee and also local professionals and stakeholders related to the research topics.

The demonstration of the effectiveness of the integrated approach is beyond the scope and the timeframe of the CIAO collaboration. The effectiveness depends largely on the capacity of local program management, involved local stakeholders, local resources, the severity/prevalence of overweight and the surrounding social and physical environment of the target population. CIAO will help to develop a better understanding of the integrated approach and offer an evaluation framework, including strategies on effectiveness, which may support local professionals in monitoring their program, taking the local context in account. An evaluation framework is important because evaluation can improve local program design which improves the likelihood of achieving successful outcomes [61].

There are multiple challenges in this type of research. CIAO researchers have to take the challenges and solutions of this type of research into account. Nastasi and Hitchcock (2009) conclude in their paper on the challenges of multilevel interventions that “even under relatively controlled experimental or quasi-experimental conditions, many factors can interfere with efforts to carry out well-designed evaluation plans” [62]. The first challenge CIAO faces is that its research depends largely on implementation efforts of the municipalities, the communities. Budget cuts or policy changes are a severe threat to CIAO research, due to a possible halt in local implementation.

The second challenge CIAO faces concerns the necessary processes and productive-interactions between the separate research teams. The integrated output of a consortium thrives on the interactions and knowledge exchange between its partners, but these interactions take time. Incentives are provided for individual research publications, but funding is only provided for five four-year research projects, and the additional requirements to establish collaboration between several research teams is not accounted for and so far not acknowledged. Simply stated, the 'glue’ between the separate research teams might be missing. Thus, for CIAO to harvest the success of the five ACCs collaborating, all stakeholders involved (executive researchers, supervisors, steering committee, supervisory committee, funders) should acknowledge that integrating group processes and competencies are essential.

The third challenge CIAO will face is 'inaction’. CIAO tries to unravel the blueprint for the integrated approach to show presumable effective elements of this approach. An important reason for this is to allow policymakers, researchers and professionals to understand the drivers and solutions of the wicked problem of overweight and obesity. However, the more thorough a description becomes, and the more it shows the complexity of the chain of causality, 'inaction’ might be the result as it raises the difficult question of where action should begin within a highly connected complex system [63].

CIAO research is important because it is the first of its sort in the Netherlands to collect solution oriented evidence in the field of overweight prevention. CIAO aims to find out what processes work best at more upstream environmental levels in an integrated community-wide approach to prevent overweight and obesity. This differs from more traditional social and behavioural sciences that try to demonstrate the efficacy of behavioural interventions to modify health outcomes. The output of the CIAO sub-studies (both knowledge and practical tools) will be matched and form building blocks of a blueprint for a local evidence- and practice-based integrated approach towards prevention of overweight in children. Subsequently, the output will support various communities to further optimize the implementation and subsequently the effects of this approach.
